# Location management for the supply of PD fluid via large neighborhood search based virus optimization algorithm

**DOI:** 10.1038/s41598-022-26385-7

**Published:** 2022-12-16

**Authors:** Walailak Atthirawong, Pongchanun Luangpaiboon

**Affiliations:** 1 Department of Statistics, School of Science, King Mongkut’s Institute of Technology Ladkrabang, Bangkok, 10520 Thailand; 2 Thammasat University Research Unit in Industrial Statistics and Operational Research, Department of Industrial Engineering, Faculty of Engineering, Thammasat School of Engineering, Thammasat University, Pathumthani, 12120 Thailand

**Keywords:** Health care, Engineering

## Abstract

The facility location problem is extended by a new two-stage zero-one programming system (2S-ZOPS). It is a type of design optimization issue that exists in logistics implementations such as supply chain planning in healthcare or agriculture. Along with concerns regarding PD delivery time manner for connecting logistics centers and customers, recent studies have considered the zero-one location design model. This research discussed a route selection model for the 2S-ZOPS that did not exist in the published studies by taking into account the level of risk associated with physical appearance. The mathematical models were developed in response to a PD supply chain design that occurred in Thailand’s National Health Insurance Program. By combining the virus optimization algorithm (VOA) with a large neighborhood search (LNS), we created a hybrid metaheuristic method for solving the 2S-ZOPS. Experiments with real-world data demonstrated that the hybrid algorithm was efficient in terms of time consumption and solution quality, saving approximately 6% on total costs. The presented practice benefits not only the healthcare industry but also various other businesses.

## Introduction

In order to provide affordable healthcare delivery, healthcare logistics costs can be reduced and efficiencies achieved. The pharmaceutical industry’s manufacturing and sales markets are currently shifting geographically. Distribution centers (or hubs) play an important role in the logistics system by connecting logistics centers and their customers to improve product flow. Appropriate distribution center locations make a substantial contribution to the efficiency of the freight system and pharmaceutical supply chains by increasing the circulation of goods and increasing the income of logistics enterprises. As a result, pharmaceutical supply chains and their third-party logistics service providers (3PLs) must better utilize and design efficient distribution networks to ensure that medicines and pharmaceutical products arrive in the right condition and quantity while minimizing system-wide costs and meeting service-level requirements^1^. Improving logistics management in healthcare organizations is becoming increasingly important in terms of cost control.

A third-party logistics (3PL) company is an external provider that controls, manages, and transports logistics services from multiple providers to multiple producers. In this study, we address the network design problem for a 3PL freight case company that is a major healthcare provider in Thailand, in order to distribute peritoneal dialysis (PD) fluid and other medical devices to public health systems in all 77 provinces of Thailand while adhering to Thailand’s universal coverage scheme. Because if PD fluids are not distributed in a timely and efficient manner, the patient’s life may be threatened, this issue is critical to investigate in this study in order to improve patient care and save lives^2,3^. At the same time, this problem should be resolved at a reasonable cost. The freight company currently has a number of storage areas and hubs spread across the country for storing and distributing healthcare products to customers^4–7^. Following production, PD fluid is aggregated and transported from the factory to storage areas and hubs before being distributed by the freight company to hospitals in each province. However, the company has never attempted to calculate the optimal number of storage and hub locations. The current study’s findings should provide some impetus to move forward in the direction of efficient nationwide management in distribution planning systems.

Prior to the delivery process, points of logistics node and transport routes from each node must be investigated and determined in order to efficiently formulate a supply chain network and support the provision of patient care^8,9^. Several companies can save transportation costs, time, and gain a competitive advantage by determining the best route. Previous route selection studies published in peer-reviewed journals used a variety of approaches and were applied to a variety of transportation products^10–14^. Since transportation infrastructure is being disrupted by natural disasters, deliberate attacks, or accidents, intelligent transportation systems (ITSs) play an important role in optimizing the route shipment problem. In the literature, using ITSs such as vehicle-to-vehicle (V2V) communication has also been considered as an alternative to finding the optimal path. V2V is a system that allows vehicles to exchange basic safety information in order to provide drivers with warnings about impending accidents, weather updates, and alternate routes in the event of traffic congestion^15^.

The majority of route selection studies have focused on multi-objective decision making based on both qualitative decision criteria like risks and quantitative decision criteria like transportation cost and time. In quality engineering, Derringer and Suich pioneered the use of more transformed desirability functions to simultaneously optimize multiple objective problems^16^. It works by converting all of the obtained objectives from different scales into a scale-free value. The values of desirability functions range from 0 to 1. The value 0 is assigned when the factors produce an undesirable objective, whereas the value 1 represents optimal performance for the factors under consideration. Based on this approach, this study used a desirability function in the form of the zero-one desirability programming (ZODP) model. The contribution is to develop a new decision-support approach for choosing the best route from each node based on quantitative and qualitative criteria for delivering PD fluid to end users.

The facility location problems have been formulated and solved mathematically using a variety of methodologies and solution techniques based on the use of various objective functions to reduce costs while improving access to healthcare services^17–19^. The literature contains a comprehensive review of healthcare location decisions^20–22^. Regardless of the research topic, the majority of the studies continued to concentrate on the development or improvement of a specific solution technique based on numerical data^23^. Their contributions to real-world problems on this subject are limited. As a result, our research was focused on a real-life scenario. The methodologies proposed in this study are generic and can be applied to other businesses with similar requirements. Many studies have emphasized the use of the FLP in various areas via exact approaches^24,25^, neural networks^26^, metaheuristics^27^ and hybrid approaches^28^.

In general, the primary goal of metaheuristic methods is to provide the best solutions in the simplest way possible in a reasonable amount of time, thereby overcoming the issues inherent in iterative simulations^29,30^. The neighborhood of achieved solution space could be developed to overcome their algorithmic limitations, particularly for large instances^31^. Virus Optimization algorithm (VOA) is one among various population-based techniques used to solve many optimization problems^32^. However, to the best of our knowledge, no VOA study has yet been proposed in FLP and healthcare products. Furthermore, several studies suggested that combining local search methods with other metaheuristics could reduce reliance on neighborhood choice while still providing flexibility and high efficiency^33–35^. To address these limitations, this paper first presents a hybridization of the large neighborhood search (LNS)^36^ and virus optimization algorithms (LNS-VOA) to address optimal storage areas and hubs for supplying PD fluid in order to obtain a reliable result.

**Figure 1:**
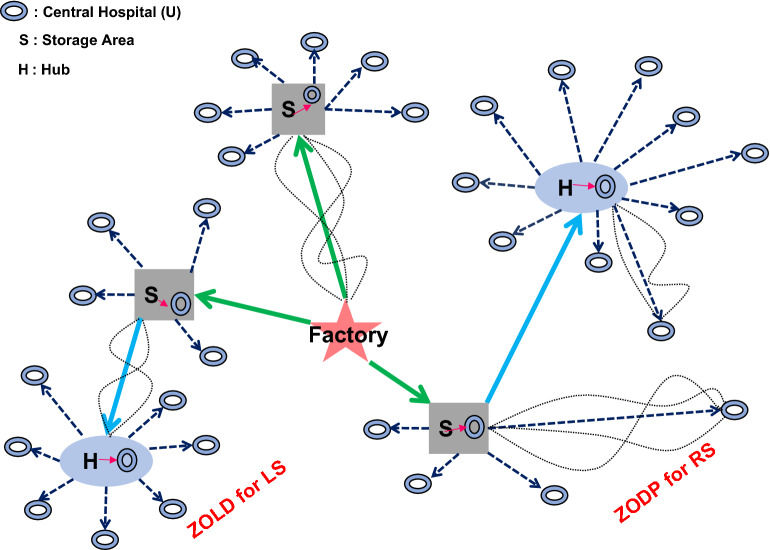
Diagram of a transportation management system for PD supplies.

This work determined the locations of PD supply storage areas and hubs (Fig. 1). The goal was to provide the freight company with the lowest possible total cost of operation. This paper’s contributions are summarized below.

1. In this paper, a new two-stage zero-one programming system (2S-ZOPS) for route and location selection is proposed.
2. To begin, zero-one desirability programming (ZODP) is used to select the best route from each hub or storage area to central hospitals or from each storage area to hubs in terms of qualitative and quantitative performance measures.
3. The center of gravity (CoG) method was introduced to address the locations of hubs and storage areas of PD fluid using all end users demand and distances from a real problem.
4. Finally, zero-one location design is used to determine the locations of hubs and storage areas (ZOLD) via the *large neighborhood search based virus optimization algorithm* and real case study is performed to validate the results.

The remainder of the paper is structured as follows. The route and location selection models are developed in the “Problem statement and formulations” section. The literature, motivation, and details about large neighborhood search and virus optimization algorithms, including hybridization, are summarized in the “Large neighborhood search based virus optimization algorithm (LNS-VOA)” section. The computational results are discussed in the section “Numerical results”. The final section is titled “Conclusions and discussions”.

## Problem statement and formulations

**Figure 2:**
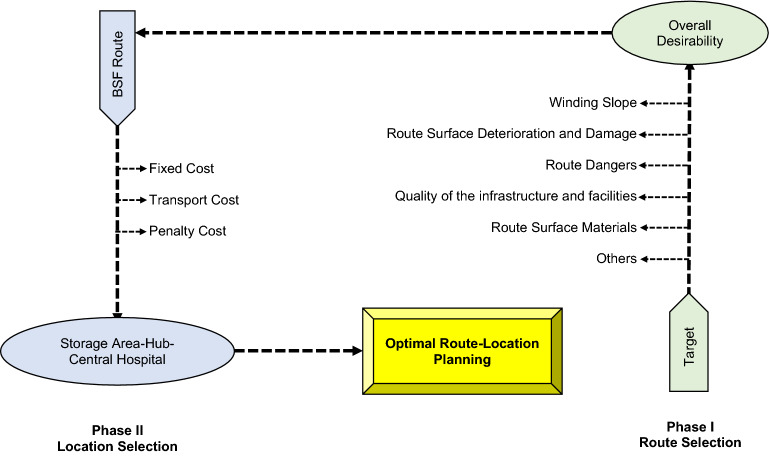
Diagram of two-stage zero-one programming system.

We focused on selecting suitable routes and storage area locations for the freight company in this study. The goal is to reduce transportation costs through the use of mathematical models via the Two-stage zero-one programming system (2S-ZOPS). The cost of transportation from the factory to distribution centers is accounted for during the manufacturing process. The number of storage areas and hubs will influence the overall cost of this mode of transportation. The two-stage zero-one programming system proposed in this study, depicted in Fig. 2, includes models for route and location selection.

### Route selection

Zero-one desirability programming (ZODP), a mathematical programming method, was firstly introduced for determining the best level for a set of decision variables with conflicting objectives. In grading techniques, a ‘1’ indicates a high benefit and a ‘10’ indicates a low benefit. The ZODP route selection model tries to increase the desired level from all objectives^37^. The *n*th route support for the *j*th objective is measured and denoted by *A*_*jn*_. Concurrent rather than sequential consideration is given to the capability of a multi-objective decision model. It seeks to improve the location selection model because clearly defining priorities is not always possible.

In Eq. (1), a geometric mean (*D*) is used to aggregate the individual desirability function (${{d}}_{{j}}$). Each objective can be simplified by converting the original data into the target differences, ${\Delta }_{{jn}}$, and its candidate values ($\sum_{{{n}}{=}{1}}^{{P}}{{{A}}}_{{jn}}{{{r}}}_{{n}}$) in the *j*th target (Eq. (2)). In Eq. (3), the converted objectives can be defined and followed by Derringer’s desirability function of minimization or smaller-the-better (STB)^38^. *D* is the minimum desirability value with respect to all the objectives (Eq. (4)). Parameters, indexes and decision variables of the ZODP model are shown in Table 1^39^.

1 $${\text{Max }}D = \left[ {\prod\limits_{{j = 1}}^{O} {d_{j} } } \right]^{{1/O}} ,$$

subject to:

2 $$\Delta _{{jn}} = \left| {O_{j}^{T} - A_{{jn}} R_{n} } \right|\;\begin{array}{*{20}l} {;\;j = 1,2,...,O} \\ {;\;n = 1,2,...,R} \\ \end{array} ,$$

3 $${D}_{{j}} = \left\{ {\begin{array}{*{20}l} {1;} \hfill & { if\, \Delta_{jn} \le R _{{j}}^{W} } \hfill \\ {\left[ {\frac{{O _{{j}}^{{T}} - \Delta_{jn} }}{{O _{{j}}^{{T}} - O _{{j}}^{W} }}} \right]^{{T_{{j}} }} ;} \hfill & { if\, O _{{j}}^{W} < \Delta_{jn} \le O _{{j}}^{{T}} } \hfill \\ {0;} \hfill & {if\,\Delta_{jn} \ge O _{{j}}^{{T}} } \hfill \\ \end{array} } \right.; \quad {j} = 1, 2, \ldots , O,$$

4 $${d}_{{i}}\ge D; \quad {j} = 1, 2, ..., O,$$

5 $${R}_{n} = 0{{\, or \,1}};\quad n = {1},{ 2},...,R.$$

**Table 1:** Parameters and decision variables for route selection, including definitions.

Parameter and decision variable	Definition
${\text{O }}_{\text{j}}^{\text{T}}$	Targeted level for objective, j
${\text{O }}_{\text{j}}^{\text{W}}$	Worst level for objective, j
A_jn_	Objective contribution, j, of route, n
A_kn_	kth system constraint contribution of route, n
${\Delta }_{\text{jn}}$	Transformed objective or level, j, towards the preferred objective level for route n (Smaller-the-Better, STB)
b_k_	kth system constraint availability
T ^j^	Parameter to adjust the shape of transformed objective, j
${\text{d}}_{\text{j}}$	Desirability function for objective, j
${\text{r}}_{\text{n}}$	nth route to be evaluated; n ∈ {1, 2, 3, …, R}
${\text{r}}_{\text{n}}\upepsilon \{\text{0,1}\}$	${\text{r}}_{\text{n}}$: 1 if the nth route is chosen; 0 otherwise

### Location selection

Our zero-one location design (ZOLD) model was therefore proposed to select high-value locations, lowering goods transport costs. Precise and reliable transport action costs and shipment expenditure are necessary to develop a mathematical model for identifying storage area and hub locations^40^. The objective function (6) is to find the storage areas and hubs locations that lead to lowest levels of overall transport cost^41^. This function includes storage area and hub operating costs, the transport costs from the manufacturer in Rayong to the storage areas; from the storage areas to the hubs, and within the province, that contains the storage areas or hubs, from the storage areas to the central hospitals or end users and from the hubs to the central hospitals or end users. It also includes penalties for failing to utilize a vehicle’s total functionality on any excursion, i.e. from the factory to storage area, *i*, from storage area, *i*, to hub, *j*, from storage area, *i*, to central hospital, *k*, from hub*, j*, to central hospital, *k*, as well as within the province containing storage area, *j*, and within the province containing hub, *j*.

The constraint (7) confirms that at least one storage area must be considered. Constraint (8) requires that customers in all provinces will receive products. Constraints (9) and (10) require that the hubs will receive from only one storage area. Constraint (11) requires that only one storage area or hub is in one province. Constraints (12) and (13) require that the central delivery points will receive products from only one storage area or hub. Constraints (14) and (15) are used for determining the number of vehicle trips required from the Rayong manufacturer to the storage areas and from the storage area to the hubs. Constraints (16) to (20) are numerical constraints on the decision variables. Our ZOLD reduces total fixed costs and transportation costs for all vehicles. Table 2 depicts all of the indices, parameters, and variables used in the ZOLD model.

6 $$\begin{aligned} {\text{MIN Z}} = & \sum\limits_{{\text{i} = 1}}^{\text{I}} {\text{C}_{\text{i}}^{\text{F}} } \text{Y}_{\text{i}} + \sum\limits_{{\text{j} = 1}}^{{18}} {\text{C}_{\text{j}}^{\text{F}} } Z_{\text{j}} + \sum\limits_{{\text{i} = 1}}^{\text{I}} {\text{C}_{\text{i}}^{\text{T}} } \text{D}_{\text{i}} \text{R}_{\text{i}} \text{Y}_{\text{i}} + \sum\limits_{{\text{i} = 1}}^{\text{I}} {\sum\limits_{{\text{j} = 1}}^{{18}} {\text{C}_{\text{ij}}^{\text{T}} } } \text{D}_{\text{ij}} \text{R}_{\text{j}} \text{X}_{\text{ij}} \\ & + \sum\limits_{{\text{i} = 1}}^{\text{I}} {\text{C}_{\text{i}}^{\text{PW}} } \text{N}_{\text{i}} \text{Y}_{\text{i}} + \sum\limits_{{\text{j} = 1}}^{{18}} {\text{C}_{\text{j}}^{\text{PW}} } \text{N}_{\text{j}} Z_{\text{j}} + \sum\limits_{{\text{i} = 1}}^{\text{I}} {\sum\limits_{{\text{k} = 1}}^{{77}} {\text{C}_{\text{ik}}^{\text{P}} } } \text{D}_{\text{ik}} \text{N}_{\text{k}} \text{X}_{\text{ik}} \\ & + \sum\limits_{{\text{j} = 1}}^{{18}} {\sum\limits_{{\text{k} = 1}}^{{77}} {\text{C}_{\text{jk}}^{\text{P}} } } \text{D}_{\text{jk}} \text{N}_{\text{k}} \text{X}_{\text{jk}} + \sum\limits_{{\text{i} = 1}}^{\text{I}} {\text{P}_{\text{i}}^{\text{T}} } + \sum\limits_{{\text{i} = 1}}^{\text{I}} {\sum\limits_{{\text{j} = 1}}^{{18}} {\text{P}_{\text{ij}}^{\text{T}} } } + \sum\limits_{{\text{i} = 1}}^{\text{I}} {\text{P}_{\text{i}}^{\text{PW}} } + \sum\limits_{{\text{j} = 1}}^{{18}} {\text{P}_{\text{j}}^{\text{PW}} } + \sum\limits_{{\text{i} = 1}}^{\text{I}} {\sum\limits_{{\text{k} = 1}}^{{77}} {\text{P}_{\text{ik}}^{\text{P}} } } + \sum\limits_{{\text{j} = 1}}^{{18}} {\sum\limits_{{\text{k} = 1}}^{{77}} {\text{P}_{\text{jk}}^{\text{P}} } } , \\ \end{aligned}$$

subject to:

7 $$\sum_{\text{i}=1}^{\text{I}}{{\text{Y}}_{\text{i}}}\ge {\text{i}},$$

8 $$\sum_{\text{i}=1}^{\text{I}}{{\text{X}}_{\text{ik}}}+\sum_{\text{j}=1}^{18}{{\text{X}}_{\text{jk}}}=1;\quad {\forall }_{\text{k}},$$

9 $$\sum_{\text{i}=1}^{\text{I}}{{\text{X}}_{\text{ij}}}{{=\text{Z}}_{\text{j}}};\quad {\forall }_{\text{j}},$$

10 $${\text{X}}_{\text{ij}}{\le \text{Y}}_{\text{i}}; \quad { \forall }_{\text{i}}{\forall }_{\text{j}},$$

11 $${{\text{X}}_{\text{ij}}}=0; \quad { \forall }_{\text{i}}={\text{j}},$$

12 $${\text{X}}_{{{\text{ik}}}} \le {\text{Y}}_{{\text{i}}} ;\quad \forall _{{\text{i}}} \forall _{{\text{k}}},$$

13 $${\text{X}}_{{{\text{jk}}}} \le {\text{Z}}_{{\text{j}}} ;\quad \forall _{{\text{j}}} \forall _{{\text{k}}},$$

14 $${{\text{R}}_{\text{i}}}\ge \frac{\left(\sum_{\text{k}=1}^{77}{{\text{Q}}_{\text{K}}}{{\text{X}}_{\text{ik}}}+\sum_{\text{j}=1}^{18}\sum_{\text{k}=1}^{77}{{\text{Q}}_{\text{K}}{}{\text{X}}_{\text{jk}}}{{\text{X}}_{\text{ij}}}\right)}{{\text{CA}}_{\text{T}}}; \quad { \forall }_{\text{i}},$$

15 $${{\text{R}}_{\text{j}}}\ge \frac{\left(\sum_{\text{k}=1}^{77}{{\text{Q}}_{\text{K}}}{{\text{X}}_{\text{jk}}}\right)}{{\text{CA}}_{\text{T}}}; \quad {\forall }_{\text{j}},$$

16 $${\text{Y}}_{\text{i}}\upepsilon \left\{\text{0,1}\right\}; \quad{ \forall }_{\text{i}},$$

17 $${\text{Z}}_{\text{j}}\upepsilon \left\{\text{0,1}\right\}; \quad{ \forall }_{\text{j}},$$

18 $${\text{X}}_{\text{ij}}\upepsilon \left\{\text{0,1}\right\}; \quad{ \forall }_{\text{i}}{\forall }_{\text{j}},$$

19 $${\text{X}}_{\text{ik}}\upepsilon \left\{\text{0,1}\right\}; \quad { \forall }_{\text{i}}{\forall }_{\text{k}},$$

20 $${\text{X}}_{\text{jk}}\upepsilon \left\{\text{0,1}\right\}; \quad{ \forall }_{\text{j}}{\forall }_{\text{k}}.$$

**Table 2:** Parameters and decision variables including their definitions for location selection.

Parameter and decision variable	Definition
*I*	Storage area index
*J*	Hub index; j ∈ {1, 2, 3, …, 18}
*K*	Central delivery point index; k ∈ {1, 2, 3, …, 77}
${\text{C}}_{\text{i}}^{\text{F}}$	Fixed cost for storage area *i*
${\text{C}}_{\text{j}}^{\text{F}}$	Fixed cost for hub *j*
${\text{C}}_{\text{i}}^{\text{T}}$	Vehicle transport cost: factory → storage area *i*
${\text{C}}_{\text{ij}}^{\text{T}}$	Vehicle transport cost: storage area *i* → hub *j*
${\text{C}}_{\text{i}}^{\text{PW}}$	Small vehicle cost within province containing storage area *i*
${\text{C}}_{\text{j}}^{\text{PW}}$	Small vehicle cost within province containing hub *j* from storage area *i*
${\text{C}}_{\text{ik}}^{\text{PW}}$	Small vehicle cost: storage area *i* → central hospital *k*
${\text{C}}_{\text{jk}}^{\text{PW}}$	Small vehicle cost: hub *j* → central hospital *k*
${\text{P}}_{\text{i}}^{\text{T}}$	Penalty for failing to utilize the entire capacity of a large vehicle: manufacturer → storage area *i*
${\text{P}}_{\text{ij}}^{\text{T}}$	Penalty for failing to utilize the entire capacity of a large vehicle: storage area *i* → hub *j*
${\text{P}}_{\text{i}}^{\text{PW}}$	Penalty for failing to utilize the entire capacity of a small vehicle within the province containing storage area *i* to central hospital *k*
${\text{P}}_{\text{j}}^{\text{PW}}$	Penalty for failing to utilize the entire capacity of a small vehicle capacity within the province containing hub *j* to central hospital *k*
${\text{P}}_{\text{ik}}^{\text{P}}$	Penalty for failing to utilize the entire capacity of a small vehicle: storage area *i* → central hospital *k*
${\text{P}}_{\text{jk}}^{\text{P}}$	Penalty for failing to utilize the entire capacity of a small vehicle : hub *j* → central hospital *k*
${\text{D}}_{\text{i}}$	BSF distance—Rayong manufacturer → storage area *i*
${\text{D}}_{\text{ij}}$	BSF distance from the storage area *i* to the hub *j*
${\text{D}}_{\text{ik}}$	BSF distance from the storage area *i* to the central hospital *k*
${\text{D}}_{\text{jk}}$	BSF distance: hub *j* → central hospital *k*
${\text{D}}_{\text{i}}^{\text{PW}}$	BSF distance: central post office → central hospital within province containing storage area *i*
${\text{D}}_{\text{j}}^{\text{PW}}$	BSF distance from the central post office to the central hospital within the province located as the hub *j*
${\text{R}}_{\text{i}}$	Number of vehicles used: Rayong manufacturer → storage area *i*
${\text{R}}_{\text{j}}$	Number of small vehicles used: storage area *i* → hub *j*
${\text{N}}_{\text{i}}$	Number of small vehicles used: transport to central hospital *k* within storage area *i*
${\text{N}}_{\text{j}}$	Number of small vehicles used: to central hospital *k* within hub *j*
${\text{N}}_{\text{k}}$	Number of small vehicles used: storage area *i* or hub *j* → central hospital *k*
${\text{Q}}_{\text{k}}$	Demand for central hospital, *k*
${\text{CA}}_{\text{T}}$	Large vehicles capacity (60 boxes throughout)
${\text{Y}}_{\text{i}}\upepsilon \{\text{0,1}\}$	${Y}_{\text{i}}$: 1 if storage area *i* is selected; 0 otherwise
${\text{Z}}_{\text{j}}\upepsilon \{\text{0,1}\}$	${\text{Z}}_{\text{j}}$: 1 if hub *j* is selected; 0 otherwise
${\text{X}}_{\text{ij}}\upepsilon \{\text{0,1}\}$	${\text{X}}_{\text{ij}}$: 1 if there is the transport storage area *i* → hub *j*; 0 otherwise
${\text{X}}_{\text{ik}}\upepsilon \{\text{0,1}\}$	${\text{X}}_{\text{ik}}$: 1 if there is the transport: storage area *i* → central hospital *k*; 0 otherwise
${\text{X}}_{\text{jk}}\upepsilon \{\text{0,1}\}$	${\text{X}}_{\text{jk}}$: 1 if there is the transport: from hub *j* to central hospital *k*; 0 otherwise

PW indicates a small vehicle, e.g. a ‘pick-up’ or ‘utility’; T indicates a large vehicle, e.g. an ‘18-wheeler’ truck.

The center of gravity (CoG) was used to determine where storage areas (S) and hubs should be placed (H). Three different scenario models were tested as potential storage area and hub locations for the freight company using three data sets of alternative locations identified.

- *Scenario A* nine storage areas in nine provinces were suggested by the freight company.
- *Scenario B* the CoG method was used to determine nine storage areas in nine provinces, with the goal of minimizing the weighted-average distance to all demand points across the country.
- *Scenario C* five locations from the scenario B set, representing each region of Thailand It determined whether the total costs of this scenario were lower than those of scenario B when the number of storage areas was reduced.

Each storage area was responsible for delivering products to hubs, center hospitals, or end users. The 77 provinces are divided into 18 groups by the Interior Ministry. As a result, the CoG method was used to select 18 alternative hub locations from those 18 province groups, with distance weighted by mass supplied or consumed. Table 3 shows the facility alternatives for storage areas and hubs that were investigated further in order to find a suitable location set in our case study.

**Table 3:** Alternative locations for the storage areas and hubs.

Alternative locations for storage areas		Alternative locations for hubs
Scenario A	Phitsanulok, Rayong, Surat Thani, Lamphun, Ubon Ratchathani, Songkhla, Nakhon Sawan, Sakon Nakhon, Roi Et	Phitsanulok, Rayong, Surat Thani, Lamphun, Ubon Ratchathani, Songkhla, Nakhon Sawan, Sakon Nakhon, Roi Et, Samut Prakan, Udon Thani, Nakhon Ratchasima, Chiang Rai, Ayutthaya, Lopburi, Nakhon Pathom, Samut Sakhon, Phuket
Scenario B	Phitsanulok, Rayong, Surat Thani Lamphun, Ubon Ratchathani, Songkhla, Samut Prakan, Udon Thani, Nakhon Ratchasima	
Scenario C	Phitsanulok, Rayong, Surat Thani, Lamphun, Ubon Ratchathani	

## Large neighborhood search based virus optimization algorithm (LNS-VOA)

The proposed algorithm is based on the VOA, a powerful population-based method that starts with a small number of viruses (solutions). At each iteration of the LNS, a portion of the current VOA solution is destroyed and reoptimized.

### Virus optimization method (VOA)

The virus optimization method (VOA) is a nature-inspired population-based metaheuristic algorithm^42^. VOA was inspired to attack a live cell by viral activity. Its behavior was compared to that of a virus infecting a cell in various natural ways. *An initialization procedure* is performed by the VOA. The researcher determines the number of initial and maximal population sizes, strong viruses, and the growth rates of strong and common viruses, as well as the initial viral population. Each virus is made up of a set of (${\text{Y}}_{\text{i}}$, ${\text{Z}}_{\text{j}}$, ${\text{X}}_{\text{ij}},{\text{X}}_{\text{ik}}{,\text{X}}_{\text{jk}}$) location selections that are generated at random and based on a set of permissible values. In *a classification procedure*, the VOA divides viruses into two categories: strong and common. The members of the strong virus (SV) were discovered to have the lowest total cost in the population. The common virus (CV) members are the remaining population of viruses whose total cost to classify as strong is higher.

After that, *a replication procedure* is used to generate new viruses (NV) with a balance of exploitation and exploration within a predetermined number of new viruses from the strong (Eq. 21) and common (Eq. 22) members. The parameters of ${\delta }^{SV}$ and ${\delta }^{CV}$ are the increments from the SV and CV, respectively. The convergence parameter of $\omega$ starts from the preset level and decrease while the mean of objective function values of the population does not improve between replication. This parameter in Eq. (22) reduces the perturbation that results in the formation of new viruses from strong members. This will allow VOA to increase exploitation in areas where there is a higher likelihood of a global optimum. Furthermore, the initial values of ${\omega \delta }^{SV}$ and ${\delta }^{CV}$ are set to be equal, implying that the perturbation for both strong and common viruses is the same in the early stages.

21 $$NV=SV \pm {\omega \delta }^{SV},$$

22 $$NV=CV \pm {\delta }^{CV}.$$

### Large neighborhood search

The LNS algorithm is based on locating the best local optima. It takes a long time to calculate to achieve a high quality result that is closer to the global optimum by searching for a large neighborhood. As a result, various techniques are employed to screen out some unnecessary neighborhood from the process. This has the potential to improve the quality of the responses. LNS will determine neighborhood through incumbent solution removal and reinsert processes. The repair and destroy functions have taken the place of the previous neighborhood search function. They specify the manner in which the search is carried out. The concept is to destroy a portion of the solution, i.e. remove it from the possible answer, and then repair it. As a result, the destroy function aims to eliminate components that allow the repair function to strengthen the solution to the greatest extent possible.

Because of the various degrees of uncertainty and ambiguity manifested in real-world applications, the goal of developing hybridization of various algorithmic ideas is to obtain better functioning and efficient formations that exploit and related advantages of the various pure strategies^43^. It starts with some basic troubleshooting to randomly select a neighborhood structure at each iteration. Following that, the search space is expanded to include more local searches than the pure VOA. To improve problem solving, destroy and repair operators are deployed during the search procedure. Operator selection is based on a weight based on previous efficiency, which is automatically updated after each local search if the new outcome is worse. The acceptance criteria will be used to decide whether to use the new solution or the previous method.

This VOA variant includes a large neighborhood search (LNS) to prevent stagnation and increase virus diversity. The main mechanism of LNS is to repeatedly destroy and repair a solution to improve it. At each iteration, two different procedures can be used to define the neighborhood to explore. The LNS1 step first looks for the location selections of the new storage areas (${\text{Y}}_{\text{i}}^{{^{\prime}}}$) with the retained members of hubs and their members or a set of (${\text{Y}}_{\text{i}}^{{^{\prime}}}$, ${\text{Z}}_{\text{j}}$, ${\text{X}}_{\text{ij}},{\text{X}}_{\text{ik}}{,\text{X}}_{\text{jk}}$). Second, it is a search for new hubs (${\text{Z}}_{\text{j}}^{{^{\prime}}}$) using the members of storage areas that have been retained or a set of (${\text{Y}}_{\text{i}}$, ${\text{Z}}_{\text{j}}^{{^{\prime}}}$, ${\text{X}}_{\text{ij}},{\text{X}}_{\text{ik}}{,\text{X}}_{\text{jk}}$) or LNS2. If the search for a complete set of (${\text{Y}}_{\text{i}}^{{^{\prime}}}$, ${\text{Z}}_{\text{j}}$, ${\text{X}}_{\text{ij}},{\text{X}}_{\text{ik}}{,\text{X}}_{\text{jk}}$) and (${\text{Y}}_{\text{i}}$, ${\text{Z}}_{\text{j}}^{{^{\prime}}}$, ${\text{X}}_{\text{ij}},{\text{X}}_{\text{ik}}{,\text{X}}_{\text{jk}}$) are successful with the better total cost and no constraint violations, run another search for the next location selection. These processes are repeated until no further exchange is possible.

**Figure 3:**
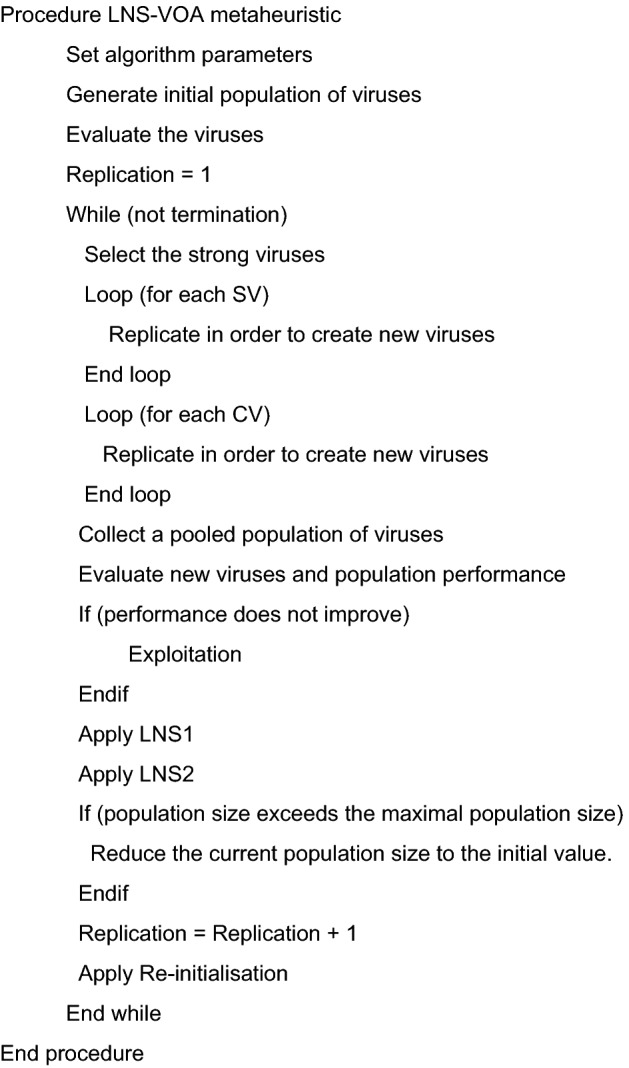
Pseudo code of LNS-VOA metaheuristic.

The exploitation mechanism groups the most powerful viruses based on their total cost. The algorithm creates new members that are more similar to stronger viruses, allowing for greater exploitation in areas where strong viruses are prevalent. The old and new viruses are then combined to form a merged population. VOA uses an anti-virus population maintenance mechanism to remove members of the population who perform poorly^44^. If a stopping criterion is not met, the replication counter increases by one and the algorithm advances to the next replication stage. Figure 3 depicts the pseudo code for the LNS-VOA.

## Numerical results

### Route selection

When it comes to pharmaceuticals, desirability includes minimizing product damage or loss, whether during transit or due to transportation time delays. The desirability of a route is influenced by the level of risk associated with its physical appearance, including population density or the number of households along the route^45,46^. Criteria for selecting the transportation route were route’s winding slope (O1), route surface deterioration and damage (O2), route dangers (O3), quality of the infrastructure and facilities (O4), materials for route surface (O5), and other factors suggested by experts (O6).

**Figure 4:**
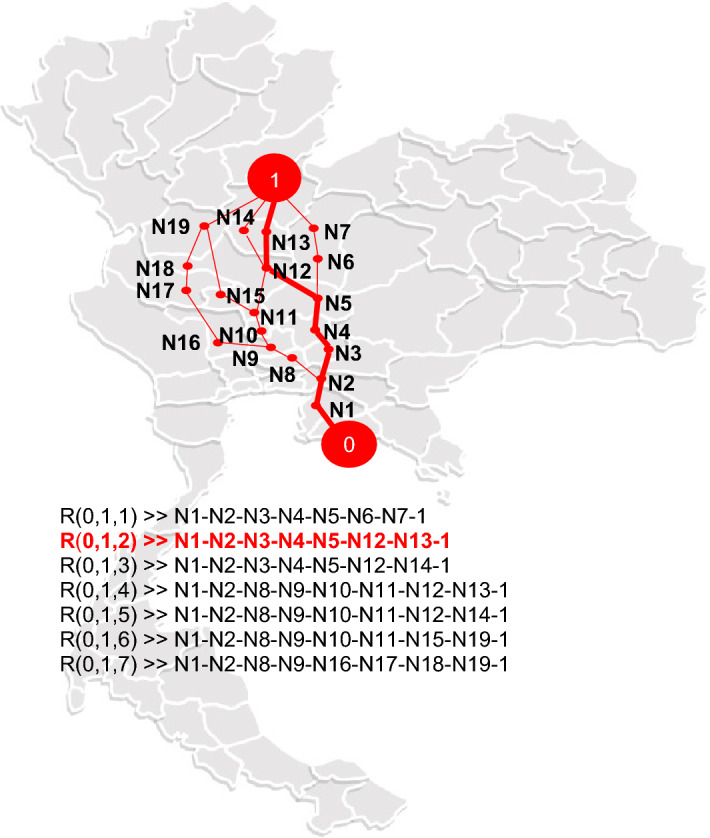
BSF route from Rayong (0) to Phitsanulok-storage area (1) with the highest D on Thailand map made with Microsoft Office 365, Excel Version 1905 (https://www.office.com/launch/excel?auth=2).

In our ZODP, the preceding section’s route selection was influenced by the highest overall desirability level. The best so far (BSF) routes from the manufacturer in Rayong to all nine storage areas and from nine storage areas based on the customer demand are shown in Table 4. Figure 4 depicts the BSF routes from the manufacturer to all storage areas in the second scenario. All figures on the Thailand map were created with Microsoft Office 365 Proplus (Version 1905) software.

**Table 4:** Overall (D), individual desirability (${\text{d}}_{\text{j}}$) function levels and BSF routes (Rt) from Rayong (0) to the 9 storage areas (S) of scenario B or R(0,S,Rt).

Storage area	R(0, S, Rt)	Desirability						
		*D*	${\text{d}}_{1}$	${\text{d}}_{2}$	${\text{d}}_{3}$	${\text{d}}_{4}$	${\text{d}}_{5}$	${\text{d}}_{6}$
Nakhon Ratchasima	R(0, 13, 1)	0.90328	0.99793	0.93946	0.90646	0.96651	0.99740	0.91605
Udon Thani	R(0, 12, 3)	0.91629	0.96848	0.99228	0.96090	0.92269	0.98869	0.96634
Samut Prakan	R(0, 11, 1)	0.93057	0.97117	0.98286	0.93555	0.95447	0.94190	0.97851
Surat Thani	R(0, 4, 3)	0.91491	0.92169	0.94151	0.99635	0.95605	0.98566	0.93059
Songkla	R(0, 5, 1)	0.90131	0.96116	0.90249	0.97700	0.96403	0.90415	0.94176
Phitsanulok	R(0, 1, 2)	0.90052	0.90192	0.93454	0.94910	0.91099	0.99049	0.95523
Lamphun	R(0, 6, 2)	0.90781	0.94831	0.98802	0.99940	0.94641	0.91400	0.91217
Rayong	R(0, 2, 5)	0.93011	0.99663	0.97236	0.95935	0.97071	0.94724	0.93362
Ubon Ratchathani	R(0, 9, 4)	0.91165	0.97103	0.98378	0.94478	0.92172	0.99310	0.91622

An optimization package that employs a zero-one programming model was used to evaluate our ZODP mathematical model. The optimization model included the concept of desirability to account for reducing overall marginal utility. Previous attempts to assess trade-offs in desirability functions focused on estimating the substitution increment between competing objectives^47^. Moving on, analyzing the impact of combining these functions into a single objective function, ‘overall desire’, is a critical task. The example used in the test was based on real-world healthcare issues.

The possible alternatives for maximizing the related objectives were heavily reliant on available routes. More notably, the analysis assisted in identifying factors that require improvement in order to achieve the BSF solution. Table 5 contains information on the BSF routes from nine storage areas to selected central hubs. Furthermore, the framework has a computational advantage because the decision maker can reduce the bias of the assessment process on each route. Based on historical data, the new conceptual framework developed probability assessment scales and intensity impact assessment measurements.

**Table 5:** Overall desirability and its BSF route (D, Rt) from all storage areas to selected central hubs.

Storage area	Hub						
	Phitsanulok	Nakorn Sawan	Samut Prakarn	Phra Nakhon Si Ayutthaya	Lopburi	Nakorn Pathom	Smut Sakorn
Phitsanulok	Na	(0.903, 2)	(0.92, 2)	(0.968, 2)	(0.990, 2)	(0.987, 5)	(0.951, 1)
Rayong	(0.967, 2)	(0.904, 2)	(0.986, 2)	(0.930, 1)	(0.926, 2)	(0.962, 2)	(0.926, 1)
RoiEd	(0.909, 2)	(0.994,1)	(0.979, 1)	(0.974, 2)	(0.930, 2)	(0.935, 1)	(0.922, 2)
Surat Thani	(0.997, 2)	(0.965,2)	(0.979, 2)	(0.986, 1)	(0.965, 2)	(0.928, 2)	(0.931, 1)
Songkla	(0.962, 1)	(0.963, 2)	(0.975, 2)	(0.945, 2)	(0.994, 2)	(0.949, 5)	0.955, 1)
Lamphun	(0.981, 1)	(0.916, 2)	(0.970, 2)	(0.924, 2)	(0.975, 2)	(0.966, 5)	(0.937, 1)
Nakhon Sawan	(0.903, 1)	Na	(0.941, 2)	(0.908, 2)	(0.973, 2)	(0.920, 5)	(0.964, 1)
Sakon Nakhon	(0.95, 2)	(0.948, 2)	(0.937, 2)	(0.976, 1)	(0.910, 1)	(0.986, 2)	(0.962, 1)
Ubon Ratchathani	(0.912, 1)	(0.923, 1)	(0.996, 2)	(0.935, 1)	(0.950, 1)	(0.984, 2)	(0.959, 1)
Chiang Rai	(0.98, 2)	(0.967, 1)	(0.997, 1)	(0.920, 1)	(0.903, 1)	(0.947, 5)	(0.940, 2)
Samut Prakarn	(0.967, 1)	(0.987, 2)	Na	(0.992, 1)	(0.919, 2)	(0.983, 2)	(0.935, 2)
Udonthani	(0.981, 1)	(0.912, 1)	(0.978, 2)	(0.961, 1)	(0.918, 3)	(0.998, 2)	(0.914, 1)
Nakhon Ratchasima	(0.927, 1)	(0.976, 3)	(0.925, 1)	(0.926, 1)	(0.981, 1)	(0.976, 1)	(0.911, 1)

The contribution of the first phase is the development of a new decision support methodology that is responsive and useful for logistics service providers in selecting transport routes based on multiple criteria. Infrastructure and facilities, the impact of accident reduction, and other factors suggested by experts are among them.

### Location selection

In this section, the LNS-VOA was implemented in Visual Basic 2018 program and each experiment was performed on an Intel Xeon E5-2650 v2 2.6 GHz CPU with 4 GB of RAM. Based on the experimental design and analysis of the parameter tuning, the important parameters for LNS-VOA of the number of strong viruses, SV generated viruses, NV generated viruses, SV increment, CV increment, $$\omega$$, maximal iterations, initial and maximal population size are 10, 10, 20, 1, 5, 5, 1000, 50, and 300, respectively. The following summarizes the relevant costs, including fixed costs of location facilities, vehicle transportation costs, and penalty costs, as well as the results of analysis using the CoG and ZOLP approaches. The average monthly cost for a new storage area was $11,500. This storage area included refrigerated storage, which was kept for subsequent delivery to hubs or directly to end users at central hospitals. Each hub served as a collection point as well as a transshipment point. The average monthly cost for the new hub was $4300.

There were two types of vehicles used: small ‘pick-up’ or ‘utility’ vehicles were used to transport products from storage areas or hubs to end users at central hospitals. A small vehicle with a loading capacity of seven boxes per trip was priced at $0.70 per kilometer. Large vehicles, which transport products from storage areas to hubs and have a load capacity of 60 boxes per trip, cost $1.10 per kilometer. According to freight company information, the transportation cost of products in the provinces chosen to establish storage areas or hubs was set at $72 on average per trip. The cost of the return trip has already been deducted.

Demands for the central hospital in each province were set to a fixed value based on the average of end-user demands collected in each province from July 2020 to December 2021. The number of end users per month for each province has been stable since the government designated the number of end users per month capable of accommodating each province’s storage capacity. The number of vehicle trips was calculated by dividing demand in each province by the number of vehicles. A penalty was declared and computed when less than the full vehicle load capacity was used. The distance from origin to destination was then calculated for use in the mathematical model, Eq. (6). Each round-trip distance from origin to destination was calculated using Google Map latitude and longitude coordinates and denoted by indices *(i,j,k)*. The calculation was divided into two phases; i.e. distance from storage area *i* to hub *j*, and from storage area *i* or from hub *j* to central hospital *k*.

There were two phases in the preliminary study to evaluate the ability of some selected nature inspired metaheuristic algorithms to deal with computational complexity issues. These algorithms consisted of firefly algorithm (FA), cuckoo optimization algorithm (COA)^14^, bat-inspired algorithm (BIA)^27^, Butterfly Optimization Algorithm (BOA), whale optimization algorithm (WOA), grey wolf optimizer (GWO)^48^ and virus optimization algorithm (VOA). The widely used public problems are solved in the first phase using non-linear mathematical test functions with single and multiple peaks, including curved ridge surfaces with a minimum dimension of two and a maximum dimension of four. Furthermore, additional experiments included single objective manufacturing optimization problems in industries^49^ in order to verify and carefully examine the entire behavior and problem solving capability for the location management of PD fluid supply. The numerical results obtained through COA, BIA and VOA demonstrated adequate accuracy in dataset compilation, which was advantageous. However, with the same stopping criterion, there was no notable change in execution time or speed of convergence.

The performance of the top three algorithms was also assessed in the second phase using three well-known multi-objective mathematical models: the multi-objective multi-pass turning model^50,51^, the complex aggregate production planning (APP) based on fuzzy model^52^, the dynamic maintenance scheduling using fuzzy data^53^ and hazardous waste management system^54^. The stopping criteria were set at 2000, 3000, and 5000 iterations. The outcomes of all three metaheuristic algorithms were evaluated using the best results obtained to date in a limited amount of time, which were obtained within 10–15 min of execution time. The VOA appears to perform better on the objective functions’ mean and standard deviation. The VOA was then used to conduct additional research on location management for PD fluid supply.

The system management contractor of National Health Insurance Program of Thailand applied Lingo previously to determine the best locations for distributing the PD supplies to central hospitals. To organize a supply chain with more position volume and other healthcare products involved, a step-by-step planning and evaluation process of optimal supply chain management, particularly route and location design^55^, is required. There is a requirement of the freight company to seek for the evaluation alternatives. Table 6 shows the details of three testing instance groups, which were created at random. Furthermore, the developed ZOLD model was used to solve three real-world scenarios in order to determine the best storage areas and hubs for the freight company’s facilities.

**Table 6:** Dataset containing nine random testing instances as well as three actual scenarios.

Index	Instance/scenario											
	S1	S2	S3	M1	M2	M3	L1	L2	L3	A	B	C
Number of storage areas	5	5	5	9	9	9	18	18	18	9	9	5
Number of hubs	12	16	18	20	22	24	32	32	32	18	18	18
Number of Central Hospitals	77	77	77	85	85	85	92	108	139	77	77	77

According to the number of storage areas, hubs and central hospitals, the instances were classified as small (S1, S2, S3), medium (M1, M2, M3), or large (L1, L2, L3). First, one province from each of the five regions—the north, north-east, south, east, and central (aside from Bangkok)—must be randomly chosen in each group of testing instances to serve as the storage area. To account for the number of storage areas in each instance, the remaining ones will also be created at random. All provinces were then chosen at random to act as hubs, whether or not they had previously served as storage areas. The computational time, which varies based on the instance size, was Lingo’s stopping criterion. The computational time was documented when attempting to find an optimal result for a small-size instance. For medium and large-size instances, the computational time was confined to 2700 and 5400 min, respectively. The experiments included Lingo and VOA, as well as the hybridization of VOA and LNS. The stopping criteria for metaheuristic algorithms were 5000 iterations.

As shown in Table 7, the numerical result was a comparison of the solutions provided by Lingo, VOA and LNS-VOA. In small instances, Lingo had the lowest total cost, and LNS-VOA appeared to be superior to VOA. In contrast, Lingo had the slowest computational time. For medium and large-size instances, Lingo was unable to determine an optimum in a reasonable amount of time. Furthermore, in terms of the BSF, LNS-VOA results outperformed the VOA at comparable computation times. Due to Scenario C’s small instance size, Lingo works best for its resolution to achieve optimality when compared to scenarios A and B. Both VOA and LNS-VOA were also able to compute to the optimal state quickly when the stopping criteria was set to 25 min. As a result, the creation of a hybrid computing system can help in a number of future scenarios. Preliminary research using the large neighborhood search algorithm suggests that developing new hybrid algorithms to improve BSF results is a viable strategy^56^.

**Table 7:** The numerical results of the testing instances and all three scenarios.

Instance/scenario	Lingo		VOA		LNS-VOA	
	Total cost ($)	Execution time (minute)	Total cost ($)	Execution time (minute)	Total cost ($)	Execution time (minute)
S1	**394,937**	203.25	445,553	**16.88**	399,708	17.02
S2	**501,899**	196.72	523,274	**17.29**	520,824	18.52
S3	**517,350**	182.94	665,487	**19.25**	518,905	20.06
M1	806,701	2700	792,431	**27.86**	**744,597**	28.23
M2	817,004	2700	803,432	28.05	**732,641**	**27.95**
M3	822,306	2700	763,688	27.48	**702,146**	**29.87**
L1	1,412,663	5400	1,394,936	**42.97**	**1,302,977**	44.34
L2	1,503,453	5400	1,452,017	44.01	**1,388,956**	**42.01**
L3	1,542,547	5400	**1,330,489**	45.04	1,368,374	**40.89**
A	693,066	2700	571,572	**20.89**	**556,811**	22.32
B	636,294	2700	583,433	**22.88**	**523,342**	23.05
C	**489,511**	186.73	552,470	**21.75**	534,240	21.83
Average	844,811	2539	823,232	**27.86**	**774,460**	28.01

Bold numbers indicate that it is the best result comparing to the results from all algorithms.

**Figure 5:**
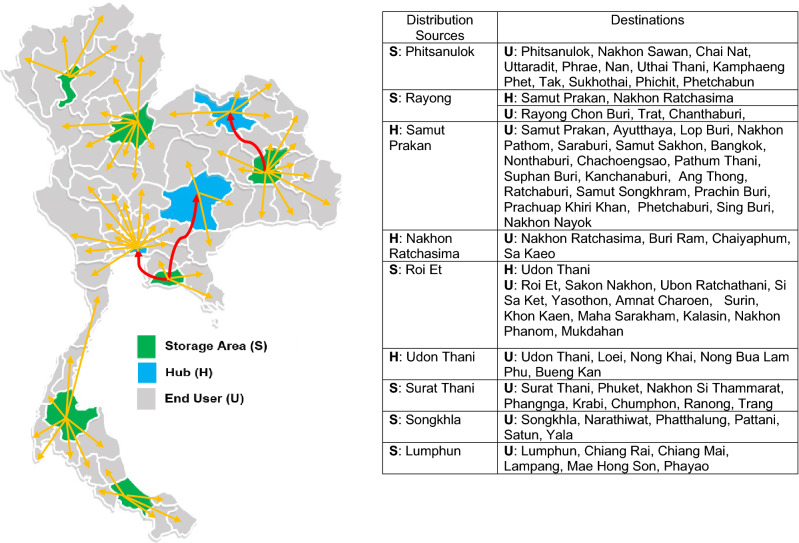
The location of facilities (storage areas or hubs) to their end users for scenario A on Thailand map created with Microsoft Office 365, Excel Version 1905 (https://www.office.com/launch/excel?auth=2).

**Figure 6:**
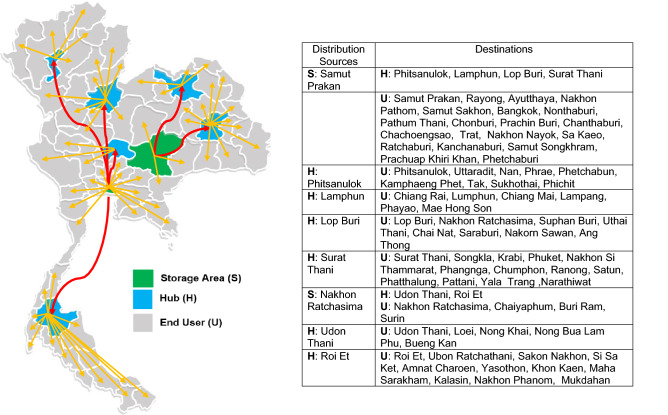
The location of facilities (storage areas or hubs) to their end users for scenario B on Thailand map created with Microsoft Office 365, Excel Version 1905 (https://www.office.com/launch/excel?auth=2).

**Figure 7:**
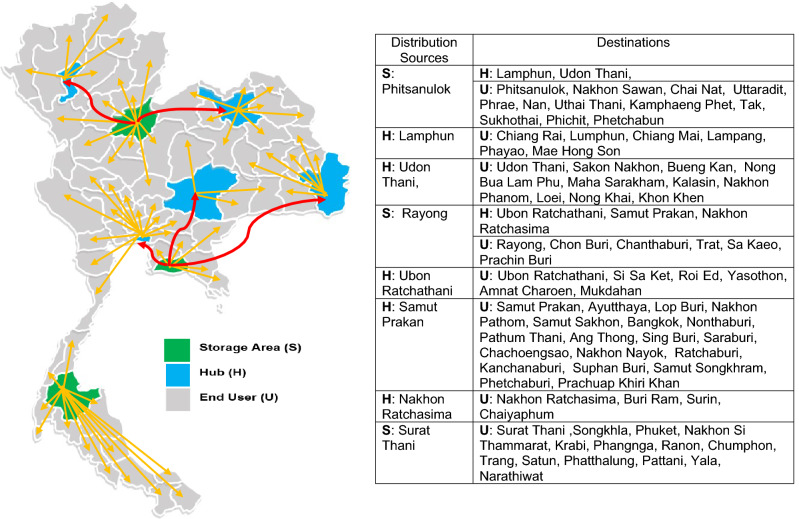
The location of facilities (storage areas or hubs) to their end users for scenario C on Thailand map created with Microsoft Office 365, Excel Version 1905 (https://www.office.com/launch/excel?auth=2).

Accordingly, the proposed LNS-VOA was selected for the third-party logistics organization to solve the case study problem of choosing the location management for PD fluid supply due to the huge time savings compared to Lingo software. Table 8 also displays the solutions for three scenarios, with details categorized by cost type. Total costs include the cost of facilities for both storage areas and hubs, as well as the cost of transportation from supply to end user demand at the central hospitals. Scenario A (Fig. 5), originally from the freight company, required $556,811 per month for six storage areas and three hubs. Scenario B (Fig. 6) required $523,342 per month for two storage areas and six hubs. Scenario C (Fig. 7) required $534,240 per month for three storage areas and five hubs. Thus, scenario B performed better than the others, followed by scenario C.

**Table 8:** Results from ZOLD model via the LNS-VOA.

Scenario	Number of facilities		Fixed cost ($)		Transport cost ($)		Penalty cost ($)	Total cost ($ )	Rank
	S	H	S	H	Large vehicles	Small vehicles			
A	6	3	68,800	12,900	210,473	260,505	4133	556,811	3
B	2	6	22,933	25,800	198,898	272,323	3388	523,342	1
C	3	5	34,400	21,500	193,754	280,374	4212	534,240	2

Figure 5 shows that, of the freight company’s existing storage areas in nine provinces, six storage areas should be chosen, which are located in six provinces, namely Phitsanulok, Rayong, Roi Et, Surat Thani, Songkhla, and Lamphun. Each storage area was responsible for distributing PD fluids directly to its own hubs and/or end users (Scenario A). For the second proposed model (Scenario B), two storage areas designed using the COG method provided the best route. Figure 6 shows that storage areas should be located in Samut Prakan and Nakhon Ratchasima provinces, while the other six hubs should be located in Phitsanulok, Lamphun, Lop Buri, Surat Thani, Udon Thani, and Roi Et provinces. Each of them distributes those products through its own hubs or directly to end users. The details of responsibility in delivering PD fluids for both storage areas and hubs was also given in Fig. 6. Finally, Fig. 7 depicts the optimal results of Scenario C, which proposed that three storage areas be located in the provinces of Phitsanulok, Rayong, and Surat Thani, with the remaining five hubs located in Lamphun, Udon Thani, Ubon Ratchathani, Samut Prakan, and Nakhon Ratchasima. The figure also includes the end users for each storage area and hub in each province. The three figures also include detailed information about their optimal route for each scenario, as well as a map of Thailand created with Microsoft Office 365, Excel Version 1905 (https://www.office.com/launch/excel?auth=2).

**Figure 8:**
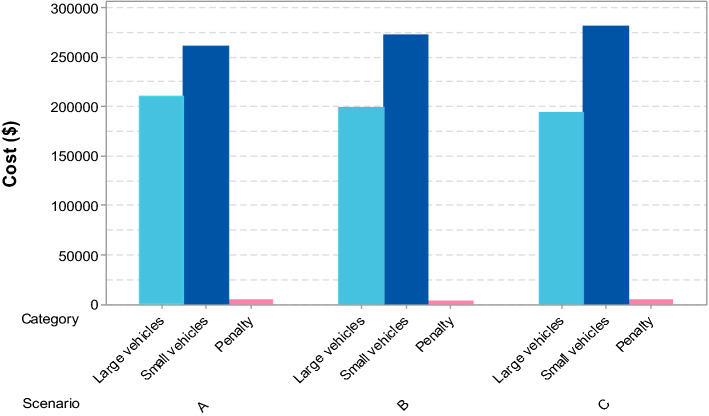
Transportation and penalty costs for the three scenarios.

Sensitivity analysis was then performed with Scenario B, increasing product demands by 15–30%, with the results revealing that the number and location of storage areas and hubs would remain unchanged. It used the zero one goal programming technique in the beginning to compare their performance. The numerical results, however, were not different when the parameter to adjust the shape of the transformed objective was set to 1. To reduce total costs, two storage areas should be located in the provinces of Samut Prakan and Nakhon Ratchasima, while the other six hubs should be located in the provinces of Phitsanulok, Lamphun, Lop Buri, Surat Thani, Udon Thani, and Roi Et. This confirmed that, in the initial step, using the CoG method to select the locations of storage areas and hubs would reduce the total cost. Figures 5, 6 and 7 show the location facilities and related end user provinces for the ZOLD model scenarios. Figure 8 compares the transportation and penalty costs of the three scenarios, revealing that scenario B has the lowest total cost.

## Conclusions and discussions

The primary goal of the healthcare supply chain within the healthcare industry is to deliver medical supplies in a timely manner to meet the needs of providers while ensuring that resources are used effectively. The industry is struggling to meet on-time delivery because of a number of issues in the distribution elements. To improve product flow, optimal logistics nodes are a critical requirement in the logistics system for connecting logistics centers and customers. As a result, positioning storage areas and hubs, as well as transportation routes, are critical activities to connect with other activities in the supply chain, which could support the efficient provision of patient care.

We created a new two-stage zero-one programming system that combined zero-one desirability programming (ZODP) and zero-one location design (ZOLD) models developed through the LNS-VOA to find optimal routes and locations for a Thai freight company that had never attempted to determine appropriate routes and storage and hub locations scattered across the country. The ZODP model was initially used to optimize transportation routes by taking into account intangible and tangible decision criteria and allowing decision makers to specify the level of risk associated with physical appearance. To account for decreasing marginal utility, the model also included the concept of desirability.

The ZODP provided a rational decision on the best so far routes, which were then used to form the ZOLD model developed via the LNS-VOA to locate storage areas and hubs in the second step. The center of gravity (CoG) method was more effective in reducing total costs to various destinations in the ZOLD initial step for determining potential storage areas and hubs. The overall total cost of the best scenario was reduced by about 6% when compared to the freight company’s traditional manual method.

The VOA metaheuristics, inspired by nature, thoroughly investigates the search space and identifies promising regions with high-quality solutions. It is ineffective at leveraging the accumulated search experiences that can be obtained by combining the LNS mechanisms with some of the permitted iterations. As a result, the resulting hybrid LNS-VOA will work in terms of complexity, at least in location management for the supply of PD fluid, by the VOA identifying promising search areas, from which the LNS can quickly determine the best so far solutions. The hybrid LNS-VOA that results from combining the strengths of both metaheuristics is frequently very effective.

Because initial parameter settings have a significant impact on solution efficiency, this approach may result in suboptimal experimental results and, as a result, a fraudulent explanation for reaching conclusions. It takes more time and effort to identify a suitable initial parameter tuning for applications with multiple metaheuristic algorithms to optimization problems. This problem requires researchers to devise a number of strategies for fine-tuning the parameters of metaheuristic methods in order to avoid a lack of motivation for the respective choices when the hybrid is used.

Extending on this research, the hybrid version of VOA and LNS can be investigated for large data and scheduling applications, discrete, combinatorial, and structural optimizations, as well as complex supply chain planning and constraint problems. Furthermore, the proposed model can be extended to address a variety of logistics system design optimization issues in healthcare and agriculture. The performance of the LNS-VOA can be improved by implementing other metaheuristic mechanisms such as genetic mutation, tabu list, and elevator random command or applying recent metaheuristics algorithms. Other approaches, such as stochastic numerical computing approaches^57–61^, or varying other parameters such as vehicle transportation costs and facility costs for both storage areas and hubs, can be used to further develop the mathematical model for routes and locations. Taking into account the uncertainty demands could be a future research topic. Other performance metrics can also be used in practical cases to validate the new model.

## Data Availability

Data available on request from the authors: The data that support the findings of this study are available from the corresponding author, [Pongchanun LUANGPAIBOON], upon reasonable request.
